# Comparison of Molecular, Clinicopathological, and Pedigree Differences Between Lynch-Like and Lynch Syndromes

**DOI:** 10.3389/fgene.2020.00991

**Published:** 2020-08-19

**Authors:** Yun Xu, Zonghao Huang, Cong Li, Congcong Zhu, Yuqin Zhang, Tian’an Guo, Fangqi Liu, Ye Xu

**Affiliations:** ^1^Department of Colorectal Surgery, Fudan University Shanghai Cancer Center, Shanghai, China; ^2^Department of Oncology, Shanghai Medical College, Fudan University, Shanghai, China; ^3^Hospital Information Centre, Fudan University Shanghai Cancer Center, Shanghai, China

**Keywords:** colorectal cancer, Lynch syndrome, Lynch-like syndrome, pedigree, DNA mismatch repair

## Abstract

In this study, we compared the molecular, clinical, and pathological characteristics, as well as pedigrees, between patients with Lynch-like syndrome (LLS) and confirmed Lynch syndrome (LS) to develop appropriate management strategies for patients with LLS and their affected family members. Between June 2008 and September 2018, 81 patients with LLS and 47 patients with LS who developed colorectal cancer (CRC) were enrolled in this study. Multigene panel testing included 139 genes and was performed for all patients. The variants identified in each group were described, and clinicopathological characteristics and pedigrees were compared between the two groups. In the LLS group, a total of 52 variants were detected in 44 (54.3%) patients. Among the 52 variants, 17 were variants of unknown significance in mismatch repair genes, and the other most frequently mutated genes were *MUYTH*, *POLE*, *BRCA2*, and *GJB2*. The proportion of early-onset patients was significantly higher among the LS probands than among the LLS probands (74.5 and 53.1%, respectively; χ^2^ = 5.712, *P* = 0.017). On the other hand, the proportion of primary CRC developed in the rectum was higher in the LLS group than in the LS group (25.9 and 10.6%, respectively; χ^2^ = 2.358, *P* = 0.046). There were no significant differences in the occurrence of metachronous CRC (*P* = 0.632) and extra-colorectal cancer (extra-CRC) (*P* = 0.145) between the two groups. However, analysis of pedigrees showed that more patients developed CRC in the LS families (*P* = 0.013), whereas more patients with extra-CRC were observed in the LLS families (*P* = 0.045). A higher prevalence of male patients was observed in the LLS families (*P* = 0.036). In conclusion, LLS should be classified as a mixed entity, containing cases of LS, other hereditary cancer syndromes, and sporadic CRC. The high risks of CRC and extra-CRCs, which were found in this study, suggest tailored management policy and surveillance should be formulated based on individual and family risk. The surveillance regimen can be based on the presence of confirmed pathogenic/likely pathogenic germline variant(s) and family history.

## Introduction

Lynch syndrome (LS) results from heterozygous pathogenic germline variants in the mismatch repair (MMR) genes that are carried by over 1 in 200 individuals (Hampel et al., 2005; Tiwari et al., 2016). Pathogenic variants in each of the MMR genes (path_MLH1, path_MSH2, path_MSH6, and path_PMS2), result in different risks for cancers in organs including the colorectum, endometrium, ovaries, stomach, small bowel, bile duct, pancreas, and upper urinary tract (Lynch et al., 2015). The consequent tumors present the phenotypes of mismatch repair (*MMR*) protein deficiency and microsatellite instability (MSI). However, there is a lack of information on pathogenic variants (PVs) in *MMR* genes for up to 50–70% of patients with *MMR*-deficient CRC tumors who were identified in population-based studies (Hampel et al., 2005; Win et al., 2015). The majority of cases in this subset are characterized by hypermethylation of the *MLH1* promoter, which is also observed in approximately 15% of sporadic CRC cases (Piñol et al., 2005; Grady and Carethers, 2008). Variants in the *BRAF* oncogene are able to distinguish LS from sporadic *MMR*-deficient CRC; this has been demonstrated to be a powerful method for screening patients with LS (Parsons et al., 2012; Boland et al., 2018). A subset of patients with CRC, who manifest the *MMR* deficiency but have no identified germline pathogenic variant in either *MMR* genes or the *BRAF* gene (absence of MLH1 methylation), have been defined as having Lynch-like syndrome (LLS) (Hampel et al., 2005; Rodríguez-Soler et al., 2013; Win et al., 2015). It has been reported that LLS may account for up to 70% of clinically suspected LS cases with a high MSI and an *MMR*-deficient profile (Carethers and Stoffel, 2015).

Molecular etiology of LLS still remains unknown, although previous findings have revealed that some groups of patients with LLS may be a mixture of LS cases, with non-detected germline variants, and sporadic CRC cases (Rodríguez-Soler et al., 2013; Carethers, 2014). Some researchers found that the risk of CRC was lower in families with LLS than in those with genetically confirmed LS (Carethers, 2014; Katz et al., 2016), while the age of CRC onset was similar for both diseases (Woods et al., 2010; Antelo et al., 2019). Nevertheless, the inability to determine the etiology of LLS hampers the development of effective screening and management policies for patients with LLS and the implementation of surveillance recommendations for these individuals and their affected relatives.

In the last decade, a wider application of multigene panel tests has provided more accurate molecular evidence for the diagnosis of LS; in the meantime, a considerable number of patients with LLS were identified at our center. Even though the genetic etiology of LLS is not defined, analyses of molecular, clinical, and pathological characteristics, as well as pedigrees of patients, may help guide decision making regarding surgical management, surveillance, and other interventions to reduce the future risks of cancer. This study was undertaken to compare the features of LS and LLS at the largest hereditary CRC research center of China, which could provide more information for the comprehensive understanding of LLS and guide management decisions for LS and LLS patients.

## Materials and Methods

### Ethics Statement

All examinations and treatments were conducted at the Fudan University Shanghai Cancer Center (Shanghai, China) and were in accordance with the Declaration of Helsinki. This study was approved by the Ethics Committee of the Fudan University Shanghai Cancer Center. Written, informed consent was obtained from the individuals for the publication of any potentially identifiable images or data included in this article.

### Patients

Between June 2008 and September 2018, a total of 139 patients with suspected LS and *MMR*-deficient profiles underwent curative surgeries, depending on the location of tumors, at the Fudan University Shanghai Cancer Centre. Multigene panel testing that included 139 genes was performed for all patients and some of their affected relatives. Informed consent for genetic analyses was obtained from all the patients. For patients with *MMR* deficiency variants in the *MLH1* or *MLH1* and *PMS2* genes, detection of *BRAF V600* variants was performed to exclude sporadic CRC.

The inclusion criteria of our study were as follows: (a) CRC confirmed by post-operative pathology; (b) *MMR* deficiency confirmed by immunohistochemistry; (c) the wild-type *BRAF V600* variant confirmed in patients without PVs in *MMR* genes. A total of 128 patients who met the inclusion criteria were enrolled in this study. Among these, 47 (36.7%) patients who were found to carry PVs in *MMR* genes were classified into the LS group, and 81 (63.3%) patients without PVs in *MMR* genes and without *BRAF V600* variants were classified into the LLS group. Carriers of variants of unknown significance (VUS) in *MMR* genes were also classified into the LLS group. Eleven patients without PVs in *MMR* genes but carrying a *BRAF* variant were excluded. Because of more than 97% concordance between the MSI and immunohistochemistry of MMR protein (Moreira et al., 2012), MSI analysis has not been performed.

### Data Collection and Follow-Up

For the 128 enrolled patients, the demographic information, pathological results, and tumor histories were retrospectively collected. The pedigrees of their families were obtained through interviews of patients and their first- and second-degree relatives, including children, siblings, parents, grandparents, aunts, and uncles. The patient and each relative were asked to report whether the relative had ever been diagnosed with cancer. For each relative, the sex of the patient, type of cancer, and age at diagnosis were recorded. Pathology documentation of cancers among relatives was systematically collected, if available.

Follow-ups were conducted for all recruited patients every 2–3 months. During the follow-up evaluation, the occurrence of metachronous CRC, distant metastases, and extra-CRC was recorded. Treatment options for these events were formulated based on the recommendations of our multidisciplinary team. Meanwhile, new cases of tumors in their families were noted, and Next-generation sequencing (NGS) was recommended for these patients. This study was censored on April 30, 2020.

### Next-Generation Sequencing

Peripheral blood (10 mL) was collected, stored in ethylenediaminetetraacetic acid tubes, and allowed to stand at 25°C for 2 h. The supernatant was transferred to a 15-mL centrifuge tube and then centrifuged for 10 min at 2,200 *g* at 4°C. Thereafter, the intermediate white blood cells were transferred to a 1.5-ml centrifuge tube. The DNA was recovered using the MagPure FFPE DNA LQ Kit (Magen). NGS was conducted on the germline DNA as a standard genetic testing for germline analysis.

DNA quantification was performed using the Qubit 2.0 Fluorimeter with the dsDNA HS assay kits (Life Technologies, Carlsbad, CA, United States). A minimum of 50 ng of DNA was required for NGS library construction. DNA shearing was performed using Covaris M220, followed by end repair, phosphorylation, and adaptor ligation. Fragments measuring 200–400 bp were selected using AMPure beads (Agencourt AMPure XP Kit, Beckman Coulter, Inc., United States), followed by hybridization with capture probes baits, hybrid selection with magnetic beads, and PCR amplification. The quality and size range of amplified fragments were then assessed by performing bioanalyzer high-sensitivity DNA assay. Paired-end sequencing of the indexed samples was performed on a NextSeq 500 sequencer (Illumina, Inc., United States).

Sequence data were mapped to the reference human genome (hg19) using BWA aligner 0.7.10. Local alignment optimization was performed using GATK 3.2. Germline SNVs were identified using Varscan with default parameters. Germline indels were identified using Varscan and GATK. Pathogenic variants were determined by a clinical molecular geneticist according to the guidelines of the American College of Medical Genetics. ClinVar and Enigma were used during manual curation for final confirmation of the results. The InSIGHT database was used for the pathogenicity classification of the *MMR* genes.

### Prediction of Pathogenicity

The pathogenicity was predicted for all detected variants using two commonly used tools, SIFT and PolyPhen2. The population frequencies of the identify variants including global and Asian frequencies were searched through The genome Aggregation Database (PRJNA398795).

### *BRAF* Variant Analysis

In all cases, surgical cancer tissues were used for the *BRAF* variant analysis. BRAF exon 15 was bidirectionally sequenced using an ABI 3730XL instrument and the BigDye Terminator v. 3.1 cycle sequencing kit (Applied Biosystems, Carlsbad, CA, United States). Three independent experiments were performed to confirm positive samples. DNA from patients was tested using the AmoyDx *BRAF* variant detection kit (Amoy Diagnostics, Xiamen, China) based on the principles of the amplification-refractory variant system. All results were confirmed according to the criteria suggested by the manufacturer.

### Statistical Analysis

Continuous variables were reported as mean ± standard deviation. Differences in categorical variables and continuous variables between these two groups were analyzed with the Chi square test or Fisher’s exact test and with Student’s *t*-test, respectively, using the SPSS version 21.0 software (SPSS, Chicago, IL, United States). Two-tailed *P*-values less than 0.05 were considered statistically significant.

## Results

### Molecular Characteristics

In the LS group, PVs of *MLH1* were identified in 17 (36.2%) probands, and those of *MSH2*, *MSH6*, and *PMS2* were identified in 18 (38.3%), 10 (21.3%), and 2 (4.2%) probands, respectively.

In the LLS group, a total of 52 variants were detected in 44 (54.3%) individuals, of which eight patients carried multiple variants. Among the 52 variants, 17 were VUS in *MMR* genes, including 8 in *MLH1*, 5 in *MSH2*, 3 in *MSH6*, and 1 in *PMS2*. Of the 17 VUS in the *MMR* genes, 8 were predicted to be possibly/probably damaging using PolyPhen2, and 4 were predicted to be deleterious using SITF. Other than *MMR* genes, the most frequently mutated genes were *MUYTH*, *POLE*, *BRCA2*, and *GJB2*. There were three biallelic missense variants in *MUYTH* (*p.Arg19Te*r, *p.Gly272Glu*, and *p.Gln267Ter*), two frameshift variants in *GJB2* (*p.His100fs* and *p.Leu79Cysfs*), and one frameshift variant in *RAD50* (*p.Glu995fs*), which were defined as pathogenic. One case of a missense variant in *BUB1B* (*p.Arg550Gln*) was defined as likely pathogenic. A total of 6 cases (9.9%) patients were confirmed carrying variants which were predispose to CRC, involving 3 (3.7%), 3 (3.7%), 1 (1.2%), and 1(1.2%) carrying variants in *MUYTH*, *GJB2*, *RAD50*, and *BUB1B*, respectively. Using PolyPhen2, 22 variants were predicted to be possibly/probably damaging, among which most were mutations in the *MLH1* and *POLE* genes. Using SIFT, 20 variants were predicted to be deleterious, among which most were mutations in the *POLE*, *MUTYH*, and *GJB2* genes. All of variants, prediction of their deleteriousness, and the frequency of each variants in globe and Asian population in the 44 patients from the LLS group are summarized in Table 1.

**TABLE 1:** Variants and prediction of deleteriousness in 44 patients of LLS group.

Gender	Age	Gene	Variants (HGVS)	Clinvar	Polyphen2^#^	SIFT*	Population frequencies (Global/Asian)
Male	60	*MLH1*	*NC_000003.12:g.37050541T*>*G (p.Val720Gly)*	VUS	Possibly damaging (0.892)	Deleterious (−5.056)	No data/no data
		*APC*	*NC_000005.10:g.112843926G*>*T (p.Ala2778Ser)*	VUS	Probably damaging (1.000)	Neutral (−0.824)	0.000156/0.00078
Male	34	*MLH1*	*NC_000003.11:g.37035002A*>*G (5 Prime UTR Variant)*	VUS	Unpredictable	Unpredictable	0.000008/0.00000
Male	55	*MLH1*	*NC_000003.11:g.37035004C*>*T(5 Prime UTR Variant)*	VUS	Unpredictable	Unpredictable	No data/no data
Male	32	*MLH1*	*NC_000003.11:g.37035057G*>*T(p.Val7Phe)*	VUS	Possibly damaging (0.485)	Neutral (−1.603)	0.00019/0.00000
Male	41	*MLH1*	*NC_000003.11:g.37035051G*>*C(p.Ala5Pro)*	VUS	Possibly damaging (0.859)	Neutral (−1.578)	No data/no data
Male	32	*MLH1*	*NC_000003.11:g.37061844A*>*G (p.Thr310Ala)*	VUS	Probably damaging (1.000)	Deleterious (−4.886)	0.000024/0.00000
Male	53	*MLH1*	*NC_000003.12:g.37012077A*>*G (p.Ile219Val)*	VUS	Benign (0.018)	Neutral (−0.460)	0.23013/0.0205
Male	46	*MLH1*	*NC_000003.11:g.37092140G*>*A (p.Arg687Gln)*	VUS	Possibly damaging (0.819)	Neutral (−1.040)	No data/no data
Male	33	*MSH2*	*NC_000001.10:g.236912509G*>*T(p.Arg534Leu)*	VUS	Probably damaging (1.000)	Deleterious (−6.708)	0.000004/0.00000
Female	29	*MSH2*	*LRG_218:g.4955G*>*A (5Prime UTR Variant)*	VUS	Unpredictable	Unpredictable	0.000024/0.00000
Female	47	*MSH2*	*NC_000002.11:g.47630246G*>*A(5 Prime UTR Variant)*	VUS	Unpredictable	Unpredictable	No data/no data
Male	37	*MSH2*	*NC_000002.11:g.47703650G*>*A (p.Ser717Asn)*	VUS	Benign (0.263)	Neutral (−2.425)	0.000004/0.00000
		*MSH2*	*NC_000002.11:g.47703539G*>*C(p.Arg680Pro)*	VUS	Probably damaging (1.000)	Deleterious (−6.534)	0.000016/0.00000
Male	61	*MSH6*	*NC_000002.12:g.47783304C*>*T(p.Ser24Leu)*	VUS	Benign (0.007)	Neutral (−0.535)	No data/no data
Female	60	*MSH6*	*NC_000002.11:g.48033801_48033825del (Splice Donor Variant)*	VUS	Unpredictable	Unpredictable	0.00002/0.00002
		*RAD50*	*NC_000005.10:g.132609343_132609346del (p.Glu995fs)*	Pathogenic	Unpredictable	Deleterious (−6.228)	0.000048/0.00016
Male	50	*MSH6*	*NC_000002.12:g.47795968C*>*T(p.Arg178Cys)*	VUS	Probably damaging (0.974)	Neutral (−1.577)	0.00006/0.0013
		*NSD1*	*NC_000005.10:g.177211614G*>*A (p.Arg1072Gln)*	VUS	Benign (0.009)	Deleterious (0.041)	0.00019/0.00000
Male	58	*PMS2*	*NC_000007.14:g.5987462G*>*A (p.His435Tyr)*	VUS	Benign (0.017)	Neutral (−0.795)	0.000004/0.00000
Female	51	*MUTYH*	*NC_000001.10:g.45800123G*>*A (p.Arg19Ter)*	Pathogenic	Unpredictable	Deleterious (−2.625)	No data/no data
		*MUTYH*	*NC_000001.10:g.45797914C*>*T (p.Gly286Glu)*	Pathogenic	Probably damaging (1.000)	Deleterious (−7.553)	No data/no data
Female	34	*MUTYH*	*NC_000001.10:g.45797972G*>*A(p.Gln267Ter)*	Pathogenic	Unpredictable	Deleterious (−9.745)	0.000016/0.00008
Female	18	*MUTYH*	*NC_000001.11:g.45334474C*>*T(p.Pro18Leu)*	VUS	Benign (0.006)	Neutral (−1.436)	0.001070/0.00537
Male	44	*MUTYH*	*NC_000001.10:g.45797760T*>*C(5 Prime UTR Variant)*	VUS	Unpredictable	Unpredictable	0.001133/0.00579
		*TP53*	*NC_000017.10:g.7579705C*>*T (p.Val31Ile)*	VUS	Benign (0.001)	Neutral (−0.142)	0.00010/0.0019
Male	25	*GJB2*	*NC_000013.10:g.20763421_20763422del (p.His100fs)*	Pathogenic	Unpredictable	Neutral (−2.065)	0.000064/0.00033
Female	47	*GJB2*	*NC_000013.10:g.20763488del (p.Leu79fs)*	Pathogenic	Unpredictable	Deleterious (−13.990)	0.000469/0.00235
Male	45	*GJB2*	*NC_000013.10:g.20763488del (p.Leu79fs)*	Pathogenic	Unpredictable	Deleterious (−13.990)	0.000469/0.00235
Female	60	*GJB2*	*NC_000013.10:g.20763150A*>*G (p.Phe191Leu)*	VUS	Probably damaging (1.000)	Deleterious (−5.719)	0.000143/0.00073
		*ATM*	*NC_000011.10:g.108250816C*>*T(p.Arg451Cys)*	VUS	Probably damaging (1.000)	Neutral (−2.171)	0.000131/0.00051
Male	54	*POLE*	*NC_000012.11:g.133215747C*>*T(p.Arg1839His)*	VUS	Probably damaging (0.993)	Deleterious (−4.652)	0.000012/0.00002
Female	51	*POLE*	*NC_000012.11:g.133225520G*>*A(p.Arg1382Cys)*	VUS	Probably damaging (0.994)	Deleterious (−5.698)	0.00003/0.0006
Female	37	*POLE*	*NC_000012.11:g.133201290C*>*T(p.Gly2285Asp)*	VUS	Benign (0.000)	Neutral (−0.437)	0.000004/0.00000
Male	64	*POLE*	*NC_000012.11:g.133250198G*>*A(p.Pro441Leu)*	VUS	Probably damaging (1.000)	Deleterious (−8.776)	0.000012/0.00002
Male	39	*POLE*	*NC_000003.11:g.37090494C*>*G(p.Pro697Ala)*	VUS	Benign (0.000)	Deleterious (−3.425)	No data/no data
Male	47	*POLE*	*NC_000012.12:g.132676107T*>*C(p.Asn336Ser)*	VUS	Probably damaging (1.000)	Deleterious (−4.472)	0.002085/0.00015
Male	61	*POLD1*	*NC_000023.10:g.152034432G*>*A (p.Gly205Ser)*	VUS	Benign (0.150)	Neutral (−0.030)	No data/no data
Male	63	*BRCA1*	*NC_000017.11:g.43106514G*>*A(p.Leu52Phe)*	VUS	Probably damaging (1.000)	Neutral (−0.587)	0.0001/0.00051
Male	68	*BRCA2*	*NC_000013.10:g.32913723G*>*T(p.Ser1744Ile)*	VUS	Benign (0.048)	Neutral (−2.182)	0.000024/0.00012
Female	44	*BRCA2*	*NC_000013.10:g.32906967G*>*A(p.Ser451Asn)*	VUS	Benign (0.009)	Neutral (−0.431)	No data/no data
Male	24	*ATR*	*NC_000003.11:g.142172064G*>*C(p.Thr2556Ser)*	VUS	Possibly damaging (0.830)	Neutral (−1.301)	0.000354/0.00180
Female	87	*EPCAM*	*NC_000002.11:g.47600631G*>*A (p.Val36Ile)*	VUS	Benign (0.002)	Neutral (−0.350)	0.000315/0.00151
Male	39	*MSH3*	*NC_000005.10:g.80813659T*>*G(p.Leu911Val)*	VUS	Benign (0.174)	Deleterious (−2.721)	0.000060/0.00031
Female	68	*PMS1*	*NC_000002.11:g.190649220G*>*A(5 Prime UTR Variant)*	VUS	Unpredictable	Unpredictable	No data/no data
Male	50	*APC*	*NC_000005.10:g.112843926G*>*T(p.Ala2778Ser)*	VUS	Probably damaging (1.000)	Neutral (−0.824)	0.000156/0.00078
		*SMAD4*	*NC_000018.10:g.51059908A*>*G (p.Asn316Ser)*	VUS	Benign (0.002)	Neutral (−0.829)	0.000056/0.00024
Female	63	*BUB1B*	*NC_000015.10:g.40202609G*>*A(p.Arg550Gln)*	Likely benign	Benign (0.001)	Neutral (0.332)	0.001646/0.00822
Female	62	*CDH1*	*NC_000016.10:g.68811716G*>*A (p.Ala289Thr)*	VUS	Probably damaging (0.932)	Neutral (−2.459	0.000004/0.00000
Female	66	*CDH1*	*NC_000016.9:g.68856080C*>*G(p.Leu630Val)*	VUS	Probably damaging (0.998)	Deleterious (−2.726)	0.000378/0.00188
Female	42	*CHEK2*	*NC_000022.10:g.29083956G*>*A (p.Arg521Trp)*	VUS	Probably damaging (1.000)	Deleterious (−4.126)	0.000051/0.00017
Male	36	*DICER1*	*NC_000014.8:g.95590896T*>*G(p.Glu338Ala)*	VUS	Benign (0.310)	Neutral (−0.642)	0.00008/0.00041

^#^These data are presented as prediction (predicted score); *these data are presented as prediction (PROVEAN score). VUS, variant of unknown significance.

The distribution of *MMR* deficiencies in the two groups was compared, and the results are summarized in Table 2. A total of 19.1% (9/46) of the patients in the LS group manifested deficiency in *MSH6* by immunohistochemistry, which was significantly higher than that (7.4%, 6/81) in the LLS group (χ^2^ = 3.963, *P* = 0.046). No significant differences were observed in case of other *MMR* deficiencies.

**TABLE 2:** Distribution of *MMR* deficiency in the two groups.

*MMR* deficiency	LS group (*N* = 47)	LLS group (*N* = 81)	χ^2^ value	*p*-value
*MLH1/PMS2*			0.482	0.487
Presence	14 (29.8%)	29 (35.8%)		
*MSH2/MSH6*			0.182	0.670
Presence	12 (25.5%)	18 (22.2%)		
Isolated *MLH1*			0.504	0.478
Presence	2 (4.3%)	6 (7.4%)		
Isolated *MSH2*			0.019	0.891
Presence	5 (10.6%)	8 (9.9%)		
Isolated *MSH6*			3.963	0.046
Presence	9 (19.1%)	6 (7.4%)		
Isolated *PMS2*			2.044	0.153
Presence	3 (6.4%)	12 (14.8%)		
Other	2 (4.3%)	2 (2.5%)	0.313	0.576
MLH1/PMS2/MSH2	1	0		
MLH1/PMS2/MSH6	1	1		
MLH1/PMS2/MSH6/MSH2		1		

**LS, Lynch syndrome; LLS, Lynch-like syndrome; MMR, mismatch repair*.*

### Demographic and Clinical Characteristics

The demographic and clinical characteristics of the 128 enrolled patients were compared between the LS and LLS groups and are summarized in Table 3. There were significant differences in the proportion of patients with the earliest onset age of CRC and in the primary CRC location between the two groups. In the LS group, 74.5% (35/47) of the patients were characterized by early-onset (<50 years old) CRC, which was significantly higher than the proportion (53.1%, 43/81) found in the LLS group (χ^2^ = 5.712, P = 0.017). In the LLS group, 25.9% (21/81) of the patients developed primary CRC in the rectum, which was remarkably higher than the proportion (10.6%, 5/47) found in the LS group (χ^2^ = 2.358, *P* = 0.046). In the comparison of the demographic and clinical characteristics between LS group and MMR VUS subset, no significant difference was found (Table 3).

**TABLE 3:** Demographic and clinical characteristics of 128 patients with colorectal cancer in the two groups.

		LLS group (*N* = 81)			MMR VUS (*N* = 17)		
Characteristic	LS group (*N* = 47)		χ^2^/*t*-value	*P*-value		χ^2^/*t*-value	*P*-value
Gender			0.985	0.321		0.062	0.803
Male	26(55.3%)	52(64.2%)			10(58.8%)		
Female	21(44.7%)	29(35.8%)			7(41.2%)		
Age (years)^*a*^	44.36 ± 11.26	48.12 ± 13.09	–1.715	0.089	44.12 ± 10.60	0.096	0.756
<50	35(74.5%)	43(53.1%)	5.712	0.017	12(70.6%)		
≥50	12(2.5.5%)	38(46.9%)			5(29.4%)		
Diagnostic criteria			4.297	0.117		0.023	0.989
Amsterdam I	11(23.4%)	9(11.1%)			4(23.5%)		
Amsterdam II	23(43.9%)	39(48.1%)			8(47.1%)		
Bethesda	13(27.7%)	33(40.8)			5(29.4%)		
CEA (ng/ml)			0.145	0.714		1.727	0.189
<5.2	7(14.9%)	15(18.5%)			5(29.4%)		
≥5.2	40(85.1%)	66(81.5%)			12(70.6%)		
Location of colorectal cancer			7.994	0.046		7.100	0.069
Right colon	18(38.3%)	32(39.5%)			8(47.0%)		
Left colon	20(42.6%)	19(23.5%)			2(11.8%)		
Rectal	5(10.6%)	21(25.9%)			5(29.4%)		
Multiple	4(8.5%)	9(11.1%)			2(11.8%)		
Multiple tumors			2.358	0.125		0.668	0.414
Occurrence	12(25.5%)	12(14.8%)			14(82.4%)		
Absence	35(74.5%)	69(85.2%)			3(17.6%)		
Tumor size^*a*^ (cm)	5.17 ± 2.61	5.11 ± 2.49	0.127	0.899	5.06 ± 1.71	0.163	0.871
Pathological classification			5.156	0.076		0.264	0.876
Adenocarcinoma	34(72.3%)	66(81.5%)			13(76.5%)		
Adenocarcinoma with partial mucinous adenocarcinoma	5(10.7%)	11(13.6%)			2(11.8%)		
Mucinous adenocarcinoma	8(17.0%)	4(4.9%)			2(11.8%)		
Differentiation			0.365	0.833		0.878	0.645
Well differentiated	1(2.1%)	3(3.7%)			0(0)		
Moderately differentiated	28(59.6%)	45(55.6%)			12(70.6%)		
Poorly differentiated	18(38.3%)	33(40.7%)			5(29.4%		
Cancerous node			1.780	0.182		0.074	0.786
Occurrence	2(4.3%)	9(11.1%)			1(5.9%)		
Absence	45(95.7%)	72(88.9%)			16(94.1%)		
Vascular invasion			0.045	0.832		0.262	0.609
Occurrence	8(17.0%)	15(18.5%)			2(11.8%)		
Absence	39(83.0%)	66(81.5%)			15(88.2%)		
Perineural invasion			0.079	0.779		0.246	0.620
Occurrence	6(12.8%)	9(11.1%)			3(17.6%)		
Absence	41(87.2%)	72(88.9%)			14(82.4%)		
T stage			0.804	0.669		3.443	0.179
T1	7(14.9%)	8(9.9%)			0(0)		
T2	8(17.0%)	13(16.0%)			2(11.8%)		
T3	32(68.1%)	60(74.1%)			15(88.2%)		
N stage			0.911	0.634		0.362	0.834
N0	34(72.3%)	53(65.4%)			11(64.7%)		
N1	9(19.1%)	17(21.0%)			4(23.5%)		
N2	4(8.6%)	11(13.6%)			2(11.8%)		
Metastasis			0.313	0.576		0.747	0.388
Occurrence	2(4.3%)	3(3.7%)			17(100%)		
Absence	45(95.7%)	78(96.3%)			0(0)		
TNM stage			1.152	0.764		2.949	0.400
I	13(27.7%)	16(19.8%)			2(11.8%)		
II	17(36.2%)	32(39.5%)			7(41.2%)		
III	15(31.8%)	30(37.0%)			8(47.0%)		
IV	2(4.3%)	3(3.7%)			0(0)		

**^*a*^These data are presented as mean ± standard deviation; other values are presented as number of patients followed by percentage in parentheses. LS, Lynch syndrome; LLS, Lynch-like syndrome; MMR, mismatch repair*.*

### Pathological Characteristics

Comparison of the pathological results showed no significant differences in the pathological TNM stage (χ^2^ = 1.152, *P* = 0.764) and differentiation of the CRC tumors (χ^2^ = 0.365, *P* = 0.833) between the two groups. The proportion of patients with mucinous CRC was 17.0% (8/47) in the LS group, which was higher than that (4.9%, 4/81) in the LLS group, whereas the proportions of patients with adenocarcinoma and partial mucinous CRC were similar between the two groups. Thus, no significant differences were observed in pathological classification (χ^2^ = 5.516, *P* = 0.076). In the comparison of pathological characteristics between LS group and MMR VUS subset, no significant difference was found. The pathological characteristics of the CRC tumors in the two groups are summarized in Table 3.

### Primary and Metachronous CRC in Probands

During the follow-up period, 34.0% (16/47) of the patients in the LS group and 38.3% (31/81) in the LLS group developed metachronous CRC, with no significant difference observed between the groups (χ^2^ = 0.229, *P* = 0.632). The period between the occurrence of primary and metachronous CRC was 28.78 ± 29.14 months in the LS group and 38.58 ± 24.89 months in the LLS group, with no significant difference being observed between the groups (*t* = −1.033, *P* = 0.108). In the comparison of tumor history between LS group and MMR VUS subset, no significant difference was found.

The mean age of cancer onset was 43.40 ± 11.17 years in the LS group and significantly higher (47.56 ± 12.99 years) in the LLS group (*t* = −2.008, *P* = 0.049). The locations of the metachronous CRC tumors were similar to those of primary CRC. In the LLS group, 38.3% (31/81) of the patients developed rectal cancer, which was markedly higher than the proportion (17.0%, 8/47) found in the LS group (χ^2^ = 6.340, *P* = 0.012). The tumor histories in the probands from the two groups are summarized in Table 4.

**TABLE 4:** Comparison of patients’ tumor histories between LS group and LLS group.

Characteristic	LS group (*N* = 47)	LLS group (*N* = 81)			MMR VUS (*N* = 17)		
			χ^2^/*t*-value	*p*-value		χ^2^/*t*-value	*p*-value
Earliest onset age of CRC (years)^*a*^	44.36 ± 11.26	48.12 ± 13.09	–1.175	0.089	44.12 ± 10.60	0.096	0.756
Total number of CRCs^*a*^	1.55 ± 0.75	1.51 ± 0.59	0.392	0.696	1.71 ± 0.77	–0.571	0.570
Metachronous CRC			0.229	0.632		0.276	0.599
Occurrence	16(34.0%)	31(38.3%)			7(41.2%)		
Right colon cancer			0.001	0.972		0.003	0.957
Occurrence	28(59.6%)	48(59.3%)			10(58.8%)		
Left colon cancer			2.505	0.113		0.004	0.949
Occurrence	30(63.8%)	40(49.4%)			11(64.7%)		
Rectal cancer			6.340	0.012		2.439	0.118
Occurrence	8(17.0%)	31(38.3%)			6(35.3%)		
Synchronous or metachronous CRC			0.118	0.874		0.205	0.651
Occurrence	20(42.6%)	37(45.7%)			8(47.1%)		
Earliest onset age of extra-colorectal cancer (years)^a^*	48.45 ± 12.68	49.79 ± 10.28	–0.345	0.732	49.57 ± 8.38	–0.205	0.840
Synchronous or metachronous extra-colorectal cancer			2.128	0.145		1.951	0.163
Occurrence	11(23.4%)	29(35.8%)			7(41.2%)		
Earliest onset age of cancer (years)^*a*^	43.40 ± 11.17	47.56 ± 12.99	–2.008	0.049	43.24 ± 10.55	0.054	0.957
Total number of cancers^*a*^	1.89 ± 1.03	1.96 ± 0.94	–0.389	0.698	2.09 ± 1.11	0.156	0.582

^*a*^These data are presented as mean ± standard deviation; other values are presented as number of patients followed by percentage in parentheses. *These data are limited to patients who developed extra-colorectal cancer. LS, Lynch syndrome; LLS, Lynch-like syndrome; CRC, colorectal cancer; MMR, mismatch repair. VUS, variant of unknown significance.

### Extra-CRC in Probands

In the LS group, 11 patients developed 15 cases of primary extra-CRC, including 5 cases of endometrial cancer, 5 cases of gastric cancer, 2 cases of small intestinal cancer, and 1 case each of ovarian, breast, and cutaneous cancer. In the LLS group, 29 patients developed 29 cases of extra-CRCs, including 8 cases of gastric cancer, 6 cases of endometrial cancer, 4 cases each of small intestinal and breast cancer, 2 cases each of prostate and ovarian cancer, and 1 case each of ureteral carcinoma, renal cancer, and pancreatic cancer. The proportions of synchronous or metachronous extra-CRC were 23.4% (11/47) in the LS group and 35.8% (29/81) in the LLS group, with no significant difference observed between the groups (χ^2^ = 2.128, *P* = 0.145). Of patients manifested *MSH6* deficiency, 2 cases (2/9, 22.2%) developed endometrial cancer, one case (1/9, 11.1%) developed gastric cancer in LS group; 2 cases (1/6, 33.3%) developed gastric cancer.

### Family Pedigrees

A total of 142 first- and second-degree relatives who developed LS-associated cancer in the LS families and 210 of those in the LLS families were enrolled in the pedigree analysis.

In the LS families, the mean number of patients who developed CRC was 3.26 ± 2.08, which was significantly higher than that (2.42 ± 1.65) in the LLS families (*t* = 2.506, *P* = 0.013). The mean earliest age of CRC onset was 37.53 ± 8.63 years in the LS families, which was significantly lower than that (44.51 ± 13.64 years) in the LLS families (*t* = −3.156, *P* = 0.002). In terms of the tumor distribution, left colon cancer was observed in 91.5% (43/47) of the LS families, which was significantly more frequent than that (70.4%, 57/81) in the LLS families (χ^2^ = 7.762, *P* = 0.005).

**FIGURE 1:**
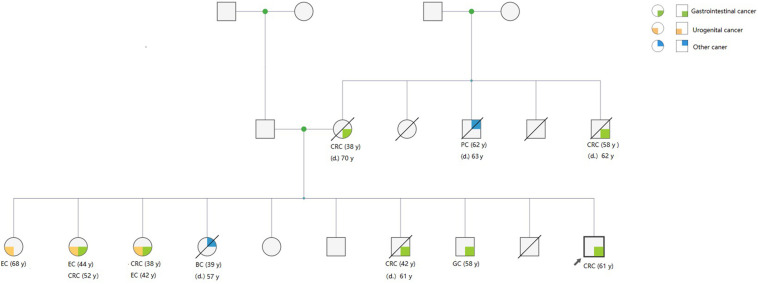
Representative pedigree of an LLS family, showing the presence of BRCA1 variants. A variant of uncertain significance in *BRCA1* (*p. Leu52Phe*) was identified in the pedigree. Three members who developed CRC underwent surgery in our hospital, and genetic testing manifested that they carried the same germline variant.

In addition to CRC, the mean number of patients who developed extra-CRC was 1.59 ± 1.38 in the LLS families, which was significantly higher than that (1.09 ± 1.37) in the LS families (*t* = −2.017, *P* = 0.045). A representative pedigree of an LLS family, demonstrating various extra-CRCs, is presented in Figure 1. Of the 10 members who developed extra-CRCs, 6 patients developed pancreatic, endometrial, breast, and gastric cancers. In the comparison of family pedigrees between LS group and MMR VUS subset, no significant difference was found. The pedigrees of the LS and LLS families were compared, and the results are summarized in Table 5.

**TABLE 5:** Comparison of pedigrees between the LS group and LLS group.

		LLS group (*N* = 81)			MMR VUS (*N* = 17)		
Characteristic	LS group (*N* = 47)		χ^2^/*t*-value	*p*-value		χ^2^/*t*-value	*p*-value
Patients with cancer (cases)^*a*^	4.02 ± 2.48	3.59 ± 1.99	1.073	0.285	3.59 ± 1.33	0.683	0.497
Male patients (cases)^*a*^	2.28 ± 1.72	2.04 ± 1.63	0.786	0.433	1.71 ± 1.31	1.244	0.218
Female patients (cases)^*a*^	1.74 ± 1.42	1.54 ± 1.32	0.808	0.421	1.82 ± 1.19	–0.204	0.839
First degree relatives (cases)^*a*^	1.98 ± 1.69	1.79 ± 1.58	0.635	0.526	1.87 ± 1.29	0.458	0.682
Second degree relatives (cases)^*a*^	1.04 ± 1.55	0.80 ± 1.23	0.968	0.335	0.91 ± 1.17	0.552	0.594
Cases of cancer^*a*^	5.13 ± 3.10	4.83 ± 2.84	0.558	0.578	5.24 ± 2.25	–0.131	0.896
Patients with CRC (cases)^*a*^	3.26 ± 2.08	2.42 ± 1.65	2.506	0.013	2.65 ± 1.41	1.114	0.270
Cases of CRC^*a*^	3.91 ± 2.54	3.12 ± 2.40	2.109	0.047	3.59 ± 2.24	0.468	0.642
Patients with right colon cancer (cases)^*a*^	1.45 ± 1.02	1.27 ± 1.13	0.877	0.382	1.53 ± 1.13	–0.279	0.781
Cases of right colon cancer^*a*^	1.49 ± 1.06	1.32 ± 1.31	0.749	0.455	1.53 ± 1.13	–0.128	0.899
Right colon cancer			1.356	0.244		0.148	0.700
Occurrence	38(80.9%)	58(71.6%)			13(76.5%)		
Patients with left colon cancer (cases)^*a*^	1.72 ± 1.19	0.98 ± 0.87	3.764	<0.001	1.18 ± 1.11	1.682	0.098
Cases of left colon cancer^*a*^	1.94 ± 1.54	1.04 ± 1.01	3.588	0.001	1.29 ± 1.11	1.577	0.120
Left colon cancer			7.762	0.005		2.949	0.086
Occurrence	43(91.5%)	57(70.4%)			12(70.6%)		
Patients with rectal cancer (cases)^*a*^	0.49 ± 0.69	0.77 ± 0.99	–1.688	0.094	0.76 ± 0.83	–1.338	0.186
Cases of rectal cancer^*a*^	0.49 ± 0.69	0.77 ± 0.99	–1.688	0.094	0.76 ± 0.83	–1.338	0.186
Rectal cancer			2.608	0.106		2.137	0.144
Occurrence	18(38.3%)	43(53.1%)			10(58.8%)		
Patients with extra-colorectal cancer (cases)^*a*^	1.09 ± 1.37	1.59 ± 1.38	–2.017	0.046	1.47 ± 1.28	–1.014	0.315
Cases of extra-colorectal cancers^*a*^	1.21 ± 1.49	1.70 ± 1.52	–2.005	0.045	1.65 ± 1.46	–1.037	0.304
Extra-colorectal cancer			1.140	0.286			
Occurrence	30(63.8%)	59(72.8%)			12(70.6%)	0.253	0.615
Synchronous or metachronous CRC			0.060	0.807		0.342	0.559
Occurrence	21(44.7%)	38(46.9%)			9(52.9%)		
Synchronous or metachronous extra-colorectal cancer			1.948	0.163		1.543	0.214
Occurrence	15(31.9%)	36(44.4%)			8(47.1%)		
Earliest onset age of cancer (years)^*a*^	36.66 ± 8.75	41.60 ± 11.91	–2.690	0.008	38.00 ± 9.31	–0.532	0.596
Earliest onset age of CRC (years)^*a*^	37.53 ± 8.63	44.51 ± 13.64	–3.156	0.002	40.35 ± 11.24	–1.064	0.292
Earliest onset age of extra-colorectal cancer (years)*	45.00 ± 10.27	47.97 ± 10.09	–1.303	0.196	48.75 ± 8.75	–1.117	0.271

**^*a*^These data are presented as mean ± standard deviation; other values are presented as number of patients followed by percentage in parentheses.****^∗^****These data are limited to families that developed extra-colorectal cancer. LS, Lynch syndrome; LLS, Lynch-like syndrome; CRC, colorectal cancer; MMR, mismatch repair; VUS, variant of unknown significance*.*

Analysis of the sex distribution showed that the mean number of the male patients in the LLS families was 2.04 ± 1.63, which was significantly higher than that (1.54 ± 1.32) of the female patients (*t* = 2.116, *P* = 0.036). In the LS families, the mean numbers of the male and female patients were 2.28 ± 1.72 and 1.74 ± 1.42, respectively, with no significant difference being observed (*t* = 1.637, *P* = 0.105).

## Discussion

With respect to oncologic outcomes, *MMR*-deficient CRC is associated with a better prognosis and therapeutic responses because the *MMR* pathway is involved in triggering cell death after chemotherapy-induced DNA damage (Gryfe et al., 2000). The prognosis in patients with *MMR*-deficient CRC tends to be better, with regard to stage-for-stage comparison, than in those with *MMR*-proficient cancer (Gryfe et al., 2000). Patients with early-stage *MMR*-deficient CRC do not appear to benefit from adjuvant 5-Fluorouracil monotherapy (Ribic et al., 2003); however, in some patients with metastatic *MMR*-deficient CRC, treatment with immune checkpoint inhibitors has been associated with an excellent response (Le et al., 2017).

However, a considerable number of *MMR*-deficient CRC tumors have an unknown etiology, other than confirmed LS and methylation of MLH1. In our study, a high proportion of patients with *MMR-*deficient CRC were diagnosed as having LLS, which was consistent with the data of a previous study (Carethers and Stoffel, 2015). Therefore, multigene panel testing should be recommended for all *MMR*-deficient patients to distinguish LS and LLS.

While management of LS has been well described, the inability to define the molecular basis of the LLS entity not only hampers the appropriate clinical management of probands, but also the cancer screening recommendations for affected families. Comparison of clinical and molecular characteristics of patients with LLS and features of their CRC tumors with those of confirmed patients with LS can contribute to the development of appropriate management recommendations for patients with LLS and their affected family members.

The genetic causes of LLS are still unknown, although advanced NGS approaches have facilitated the discovery of novel genetic events that may allow the definition of clinical and molecular phenotypes of LLS. In our study, variants were unidentified in nearly half of the LLS cohort. Current techniques of analysis may be missing complex or cryptic variants in *MMR* genes, and some deep intronic variants may be overlooked (Clendenning et al., 2011; Morak et al., 2011). Furthermore, there may be some unidentified variants in the regulatory regions of *MMR* genes, which are hardly screened (Liu et al., 2016). Thus, we suggest that this subset may have been a mixture of patients with LS, whose germline variants were not detected, and those with sporadic CRC. Future advances in NGS techniques may allow obtaining more accurate genetic information for discriminating between patients with LS and LLS.

Among the variants identified in this study, the largest category was VUS in *MMR* genes. The classification of these patients is still uncertain, and they were grouped as patients with LLS in the current study. Through comparison of this subset with confirmed LS, no significance was found in both clinical phenotypes and genealogical characteristics. Thus, some of the patients carrying VUS in *MMR* genes may have been patients with LS, which was supported by a high frequency of metachronous CRC. The pathogenicity of these VUS should be confirmed in functional experiments. The high frequency of metachronous CRC observed in our study suggests that patients with LLS should be considered high-risk cases, and tailored strategies cancer prevention which formulated based on individual and family risk must be implemented for this group of patients and their relatives (Gupta et al., 2019). Furthermore, the current management guidelines for LS should be revised in light of the genotypes, associated phenotypes, and specified cancer risk.

In addition to *MMR* genes, most of the other PVs and likely PVs were detected in the *MUTYH* and *GJB2* genes. Biallelic *MUTYH* variants have been detected in 1.8–3.1% of patients with LLS (Castillejo et al., 2014; Morak et al., 2014). *MUTYH*-associated polyposis is extremely variable, ranging from severe polyposis coli to attenuated forms with a late age of onset or few adenomas, or CRC, which creates a phenotypic overlap with LS (Morak et al., 2010, 2014). *GJB2* encodes a gap junction protein, also known as connexin 26. Variants in this gene are responsible for as much as 50% of prelingual, recessive deafness (Smith et al., 2005). The cytoplasmic Cx26 protein has been associated with the tumor progression and a poor prognosis in patients with breast cancer and esophageal squamous cell carcinoma (Naoi et al., 2007; Inose et al., 2009). To the best of our knowledge, this is the first study to demonstrate the involvement of *GJB2*, as a novel candidate gene, in LLS-linked CRC. The pathogenicity of the frameshift variant in *GJB2* is being evaluated by functional analysis, and the results will be reported separately. Variants in the exonuclease domain of the polymerase proofreading genes *POLE* and *POLD1* cause polymerase proofreading-associated polyposis, which is a dominant-inheritance and high-penetrance hereditary syndrome conferring a predisposition to attenuated colorectal polyposis and early-onset CRC (Palles et al., 2013). The association between variants of polymerase proofreading genes and *MMR* deficiency has been reported previously (Jansen et al., 2016). In our study, VUS in the *POLE* and *POLD1* genes were predicted to be deleterious and were among the most frequently detected variants. Some other variants were identified in *BRCA1*, *BRCA2*, and *RAD50*, which are involved in the homologous recombination pathway. Defects in the *BRCA* genes are known to be pathogenic causes of hereditary breast and ovarian cancers (Llort et al., 2015), in addition to conferring a high risk of developing CRC (Mersch et al., 2015).

Therefore, it is possible that some cases of LLS can be due to the pleiotropism of certain gene variants, manifesting as genetic overlaps with other hereditary cancer syndromes. Because of the mixture, a higher prevalence of extra-CRCs and a lower prevalence of CRCs were revealed in the LLS families. The high risk of extra-CRCs found in our study suggests that tailored surveillance policies of other organs should be recommended for probands and their affected family members. The surveillance regimen can be based on the presence and confirmed pathogenic variant and family history. For example, gastroduodenoscopy should be regularly performed in patients carrying *MUTYH* variants, while gynecological and breast examinations would be recommended for patients carrying *BRCA* variants. Furthermore, functional analysis of the undefined variants found in patients with LLS should be performed to elucidate the underlying molecular etiology of LLS.

The difference in the age at onset of CRC between patients with LS and LLS remains controversial; some studies demonstrated similar proportions of early-onset patients in the LS and LLS groups (Antelo et al., 2019), whereas one report showed that the population of patients with LLS was older (Porkka et al., 2019). Our results supported the latter findings, with age differences being manifested in both probands and related family members. Variants in genes such as *POLE* and *BRCA*, which were found in patients with LLS, may confer a higher risk of CRC; however, these variants show moderate penetrance (Yurgelun et al., 2017). Because sporadic CRC is combined with moderate penetrance of other variants, a delayed onset of CRC was demonstrated in probands with LLS. It is noteworthy that more than half of the patients in the LLS group were early-onset cases, which is significantly higher than the reported rate of sporadic CRC (Siegel et al., 2017). Therefore, MSI and multigene panel testing should be recommended for the early-onset subset, and screening colonoscopy at an early age should be performed in affected family members.

In terms of the CRC localization, our study showed a striking clustering of tumors in the rectum of probands with LLS, indicating that the rectum as the preferred organ can be described as a clinical feature of LLS-associated CRC. A higher frequency of left colon cancer was consistent with the findings of our previous study, which investigated clinical features of LS in an Asian population (Liu et al., 2014). While LS-associated CRC is characterized by mucinous differentiation (Llor et al., 2005), a reasonably lower proportion of mucinous tumors was observed in the LLS cohort in this study. Another interesting finding was a larger number of male patients in LLS families. A higher prevalence of male patients in LS families was reported in a previous review (Sehgal et al., 2014), but has not been previously described in LLS families. This discovery of the sex-dependent tendency of disease in LLS families may be described as clinical features of LLS.

There are some limitations of our study. First, MSI testing was not performed, the BRAF mutation detection was first performed with Sanger sequencing, which has a relative low sensitivity and no MLH1 methylation analysis was performed. Secondly, MMR deficiency can be caused due to somatic mutations in MMR genes not analyzed which may have resulted in an incorrect interpretation of the molecular evidence. Thirdly, the sample size needs to be increased, and a long-term follow-up is required. Lastly, functional experimental for some variants in the current study is still in process, and the results will be reported in subsequent articles.

In view of the limitations of this study, the enrollment of a larger cohorts and functional assays to verify the pathogenicity will be considered as priority in our subsequent research. As far as the findings of this paper were concerned, LLS should be classified as a mixed entity, containing cases of LS, other hereditary cancer syndromes, and sporadic CRC. The high risks of CRC and extra-CRCs, which were found in this study, suggest tailored management policy and surveillance should be formulated based on individual and family risk. The surveillance regimen can be based on the presence of confirmed pathogenic/likely pathogenic germline variant(s) and family history. The preference for CRC development in the rectum and higher prevalence of male patients discovered for the first time in LLS families may be described as clinical features of LLS.

## Data Availability Statement

The datasets for this article are not publicly available due to concerns regarding participant/patient anonymity. Requests to access the datasets should be directed to the corresponding author.

## Ethics Statement

The studies involving human participants were reviewed and approved by Fudan University Shanghai Cancer Center. The patients/participants provided their written informed consent to participate in this study. Written informed consent was obtained from the individuals for the publication of any potentially identifiable images or data included in this article.

## Author Contributions

YuX, YeX, and FL conceived and designed the study. YuX, ZH, CL, YZ, CZ, and TG collected and analyzed the data. YuX and ZH provided statistical expertise and were involved in data analysis and interpretation of results. YuX wrote the manuscript. CL, YeX, and FL reviewed the manuscript. All authors contributed to the article and approved the submitted version.

## Conflict of Interest

The authors declare that the research was conducted in the absence of any commercial or financial relationships that could be construed as a potential conflict of interest.
